# The Huge Reduction in Adult Male Mortality in Belarus and Russia: Is It Attributable to Anti-Alcohol Measures?

**DOI:** 10.1371/journal.pone.0138021

**Published:** 2015-09-16

**Authors:** Pavel Grigoriev, Evgeny M. Andreev

**Affiliations:** 1 Max Planck Institute for Demographic Research, Rostock, Germany; 2 New Economic School, Moscow, Russia; Cardiff University, UNITED KINGDOM

## Abstract

**Background and Aim:**

Harmful alcohol consumption has long been recognized as being the major determinant of male premature mortality in the European countries of the former USSR. Our focus here is on Belarus and Russia, two Slavic countries which continue to suffer enormously from the burden of the harmful consumption of alcohol. However, after a long period of deterioration, mortality trends in these countries have been improving over the past decade. We aim to investigate to what extent the recent declines in adult mortality in Belarus and Russia are attributable to the anti-alcohol measures introduced in these two countries in the 2000s.

**Data and Methods:**

We rely on the detailed cause-specific mortality series for the period 1980–2013. Our analysis focuses on the male population, and considers only a limited number of causes of death which we label as being alcohol-related: accidental poisoning by alcohol, liver cirrhosis, ischemic heart diseases, stroke, transportation accidents, and other external causes. For each of these causes we computed age-standardized death rates. The life table decomposition method was used to determine the age groups and the causes of death responsible for changes in life expectancy over time.

**Conclusion:**

Our results do not lead us to conclude that the schedule of anti-alcohol measures corresponds to the schedule of mortality changes. The continuous reduction in adult male mortality seen in Belarus and Russia cannot be fully explained by the anti-alcohol policies implemented in these countries, although these policies likely contributed to the large mortality reductions observed in Belarus and Russia in 2005–2006 and in Belarus in 2012. Thus, the effects of these policies appear to have been modest. We argue that the anti-alcohol measures implemented in Belarus and Russia simply coincided with fluctuations in alcohol-related mortality which originated in the past. If these trends had not been underway already, these huge mortality effects would not have occurred.

## Introduction

Excessive alcohol consumption has long been recognized as being the major determinant of male premature mortality in the European countries of the former USSR (FSU). The results of a large number of national-level studies have repeatedly suggested the existence of the strong correlation between changes in alcohol consumption and fluctuations in mortality.[1–10] A number epidemiological studies conducted in several cities of Russia have demonstrated that working-age mortality is particularly strongly associated with hazardous patterns of alcohol consumption.[11–13] Previous research has also shown that alcohol is not only associated with violent mortality [14] and mortality from liver cirrhosis, [15] but also with mortality from cardiovascular diseases.[11,16–18]

We focus on Belarus and Russia, two Slavic countries which currently suffer enormously from the burden of harmful alcohol consumption. According to the most recent WHO report on alcohol consumption, Belarus had the world’s highest total (recorded and unrecorded) level of alcohol consumption, of 17.5 liters consumed per capita in 2010.[19] According to the same report, Russia had the fourth-highest level in 2010, of 15.1 liters per adult aged 15 or older.[19] Both countries were assigned the highest YLL score, an estimate of *years of life lost* due to alcohol. In both countries, the average male aged 15 or older consumes more than 30 liters of pure alcohol a year. Together with Ukraine, Russia has the most risky patterns of drinking in the world.[19] The WHO assigned Russia the highest pattern of drinking score (PDS), or five; and gave Belarus a PDS of four [19]. There are important similarities and differences between the two countries in terms of the composition of alcohol consumption by type. In both Belarus and Russia in 2010, about a half of all of the alcohol consumed was in the form of spirits.[19] However, in Russia 38 percent of the alcohol consumed was in the form of beer and 11 percent was in the form of wine; whereas the corresponding figures for Belarus were 17 percent and five percent.[19] Meanwhile, 31 percent of alcohol consumption in Belarus in 2010 was in the form of a residual category of beverages which includes cheap fruit wines; whereas in Russia almost none of the alcohol consumption fell into this category.[19]

If we consider the long-term evolution of adult mortality and trends in alcohol-related causes of death, the situation in Belarus looks even more unfavorable than the situation in Russia. While it was a republic of the USSR, Belarus had one of the lowest mortality levels across the Soviet Union.[20] Prior to the dissolution of the USSR, the levels of alcohol-related mortality were considerably lower in Belarus than in Russia. This gap was reflected in the different effects Gorbachev’s anti-alcohol campaign had on the two republics: between 1984 and 1987, male life expectancy increased by 2.2 years in Belarus, compared to three years in Russia.[21] After the dissolution of the USSR, all-cause mortality and mortality from accidental alcohol poisoning (a known proxy for hazardous alcohol consumption [4,5]) was growing continuously in Belarus, whereas in Russia there was an abrupt increase followed by a decline.[20] Between 1986 and 2003, male life expectancy decreased 6.3 years in Russia and 5.1 years in Belarus.[21]

However, since 2003–2004 positive changes in mortality dynamics have been observed in both countries. Between 2003 and 2013 male life expectancy increased by 6.6 years in Russia [22] and by 4.6 years in Belarus.[21] In Russia, the greatest reduction in mortality was among the working-age population, and a large share of this decline was again likely attributable to the decrease in alcohol consumption.[23,24] In Belarus, a significant reduction in mortality was observed between 2011 and 2012. Within just a single calendar year male life expectancy increased by two years [21]—an improvement which is almost equivalent to the effect observed during Gorbachev’s anti-alcohol campaign.[20] In 2011, Belarusian authorities launched a massive anti-alcohol campaign.[19] Thus, changes in alcohol consumption have likely contributed to recent declines in mortality in Belarus as well.

In this paper we investigate to what extent the recent declines in adult mortality in Belarus and Russia are attributable to the anti-alcohol measures implemented in these countries. The study is organized as follows. First, as background information we provide an overview of the anti-alcohol measures adopted in Belarus and Russia during the Soviet and the post-Soviet periods. We then conduct a comparative demographic analysis of the observed mortality declines in the two countries, linking these decreases to anti-alcohol measures. Finally, we discuss the potential mechanisms underlying these rapid and somewhat unexpected mortality changes.

## Background: Alcohol Policies and Anti-Alcohol Measures

### Soviet Period

Even though excessive alcohol consumption is widely recognized as constituting a major threat to public health, the anti-alcohol policies of Belarus and Russia have generally been irregular and lacking in consistency. In the past, little was done to curb the levels of alcohol consumption in these countries. Excessive drinking was even encouraged, as alcohol production was a very profitable industry for the Soviet government.[25]. There were, however, some intermittent attempts to restrict alcohol consumption. In 1958 the Soviet government issued its first post-war directive on alcohol use: “On strengthening the fight against alcoholism and restoring order in sales of strong spirits.” Later, on May 16, 1972, Soviet authorities issued another directive: “On measures to strengthen the fight against drunkenness and alcoholism.” This directive mandated an increase in prices, a restriction in the hours of sale of alcohol (from 11 am to 7 pm), the establishment of a network of occupational therapy detox centers known as LTPs, a diversification of alcohol production (the production of less vodka but of more wine and beer), and the suspension of the production of vodka with an ethanol content of more than 40 percent. However, these measures did not have a noticeable impact on mortality. On April 1, 1982, the prices on vodka and on most other alcoholic beverages were increased, with the minimum price of vodka rising by 50 percent. In the same year, life expectancy among Russian males increased by six months.[26]

Gorbachev’s anti-alcohol campaign appears to have been far more effective than previous efforts by the Soviet government to limit alcohol consumption. The campaign included a number of extreme administrative measures, such as rationing (e.g., one bottle of vodka per adult per month), restricted hours of sale of alcohol (from 2 pm to 7 pm), and the detainment of publicly intoxicated individuals in a *Vytrezvitel* (drunk tank). There was also an absolute ban on alcohol consumption in public places and institutions, including in sports venues and on public transportation. In addition, the government developed a network of *LTPs*. The campaign resulted in a rapid reduction in mortality, especially among adult males.[5] However, because of its excessive reliance on administrative measures, the campaign did not have long-term effects, and the mass consumption of alcohol resumed immediately after the collapse of the USSR.[9,27]

### Post-Soviet Period

In the years immediately following the dissolution of the USSR, there were almost no anti-alcohol policies in the republics, and the power of the newly independent states to control the production and circulation of alcohol was very weak. These factors contributed to sharp rise in alcohol consumption, and particularly of illegal forms of alcohol, including of beverages which were manufactured locally or imported from abroad. The situation was particularly severe in Russia, where the state alcohol monopoly was abolished in 1991 and a portion of the alcohol industry was privatized.[25] Meanwhile, in Belarus the state monopoly on alcohol production was preserved. Since the mid-1990s several normative acts regulating the production, the circulation, and the consumption of alcohol have been adopted in Belarus and Russia. The list of major legislative acts, along with a description of the anti-alcohol measures, appear in the appendix (S1 Appendix).

#### Belarus

In July 1998, Belarus approved a law regulating the production and circulation of ethyl alcohol and alcohol products. Under the law, the state was given the authority to regulate not just the production, sale, and import and export of alcohol; but also alcohol prices. In August 1999, excise stamps for marking alcohol beverages were introduced in Belarus. In 2000, the Belarusian government adopted the concept of a state anti-alcohol policy, as well as a national program of actions to prevent alcoholism for 2001–2005. Presidential decrees issued in 2002 and 2005 mandated the further strengthening of state control over the production, circulation, and advertising of alcohol. In 2006, a second national program of actions to prevent alcoholism for 2006–2010 was announced. In 2008, a new law on the state regulation of the production and circulation of ethanol-containing beverages went into force, replacing the analogous law of 1998.

In 2011, a third state program of actions to prevent alcoholism for 2011–2015 was adopted in Belarus. This most recent program has several features that distinguish it from the two previous campaigns. The current program puts more emphasis on the prevention of alcoholism through the reduction of major risk factors, and has two main targets. The first target is to reduce alcohol-related mortality, and especially accidental poisoning by alcohol. The Ministry of Health is responsible for the monitoring of the progress made in reaching this target, and for improving narcology services across the country, including both the treatment and the prevention of alcoholism. The second target is the prevention of alcohol-related crimes, which is the responsibility of the Ministry of Internal Affairs. In addition to the measures undertaken in the previous programs, the 2011 anti-alcohol campaign included a number of new initiatives, such as increases in the excise duties on specific alcohol products, increases in the policing of and financial penalties on home producers, and a strengthening of laws on driving while intoxicated. The WHO has called the Belarusian anti-alcohol campaign of 2011 “an example of leadership and commitment to reducing harmful use of alcohol”.[19]

#### Russia

The alcohol policies implemented in Russia have been described in detail by a number of authors. [25,28–31] The mid-2000s is considered to be the major turning point in Russian alcohol policies. In May 2005, with the creation of the state unitary enterprise Rosspirtprom, the Russian government asserted a monopoly over the alcohol industry. A federal law signed on July 21, 2005, is the core legislative act which regulates the production and sale of ethyl alcohol and alcohol-containing products in Russia. The aim is to strengthen state control over the volume of alcohol production and sales, as well as the quality of alcohol products. Specifically, the goal is to make it more difficult to sell low-quality and illegal alcohol products in Russia. In practice, however, the implementation of new alcohol policies has been hindered in a number of ways, such as by the shortage of excise stamps and the poor utilization of the EGAIS, or the unified state automatic information system.[25]

In early 2010 the Russian government approved a national program of actions to reduce alcohol-related harm and prevent alcoholism among the Russian population for the period 2010–2020. This initiative has, however, so far been purely symbolic. As the initial estimates of alcohol consumption in Russia were significantly overstated, the Minister of Health was able to claim to claim that the main tasks of the concept had been quickly implemented.[32]

Measures to limit the consumption of non-beverage alcohol appear to be the most successful of the anti-alcohol initiatives implemented in Russia. Leon and colleagues [12] showed that there is a strong direct association between mortality and the frequency of the consumption of non-beverage alcohol. These measures not only mandated the use of denaturants in the production of liquids containing non-beverage alcohol; they also included strict controls over ethanol production at all stages. However, the most significant reduction in alcohol-related deaths, which occurred in the summer of 2006, was largely attributable to poor preparation for the introduction of new rules on the production and sale of alcohol. According to Nemtsov [33], efforts by the authorities to streamline ethyl manufacturing actually resulted in a profound disruption of the alcohol market. This disruption, along with the ban on imports of wine from Georgia and Moldova, led to a shortage of cheap alcohol, which in turn had immediate effects on mortality.[33]

## Data and Methods

For Belarus, we rely on the mortality series by sex, five-year age group, and cause of death; harmonized using the uniform method of a posteriori reconstruction of continuous mortality series.[34,35] Mortality series classified in accordance with the abridged versions of the ICD–10 are available for Belarus for the period 1965–2010. To extend the series up to 2013, we used the unpublished statistical tables by causes of death. These official data were obtained directly from the National Statistical Committee of Belarus. Age- and cause-specific mortality rates were computed on the basis of population estimates extracted from the Human Mortality Database. [21] The respective data for Russia were obtained from the Russian Fertility and Mortality Database.[36]

We focus on males aged 20–64 years. This population group has been identified as the main one in terms of the impact of alcohol consumption on mortality [37]. Our analysis does not include female mortality because it is known to be affected by alcohol consumption to a much lesser extent compared to male mortality. [38,39]

In our analysis we consider only a limited number of causes of death which we label as being *alcohol-related*: namely, accidental poisoning by alcohol (corresponds to item X45 in the ICD–10), cirrhosis of the liver (K70, K74), ischemic heart diseases (I20–I25), stroke (I60–I67), transportation accidents (V02–V99), and other external causes (X–Y except X45, V02–V99). Although there are many more causes of death which can be linked to alcohol, we do not consider them in the analysis due their very small contributions to mortality.

To ensure the comparability of mortality trends, we calculated age-standardized death rates (SDRs) on the basis of the European population standard.[40] To determine the particular age groups and the causes of death responsible for changes in life expectancy over time, we used the life table decomposition method.[41]

## Results

### Trends in Alcohol-related Causes

**Fig 1:**
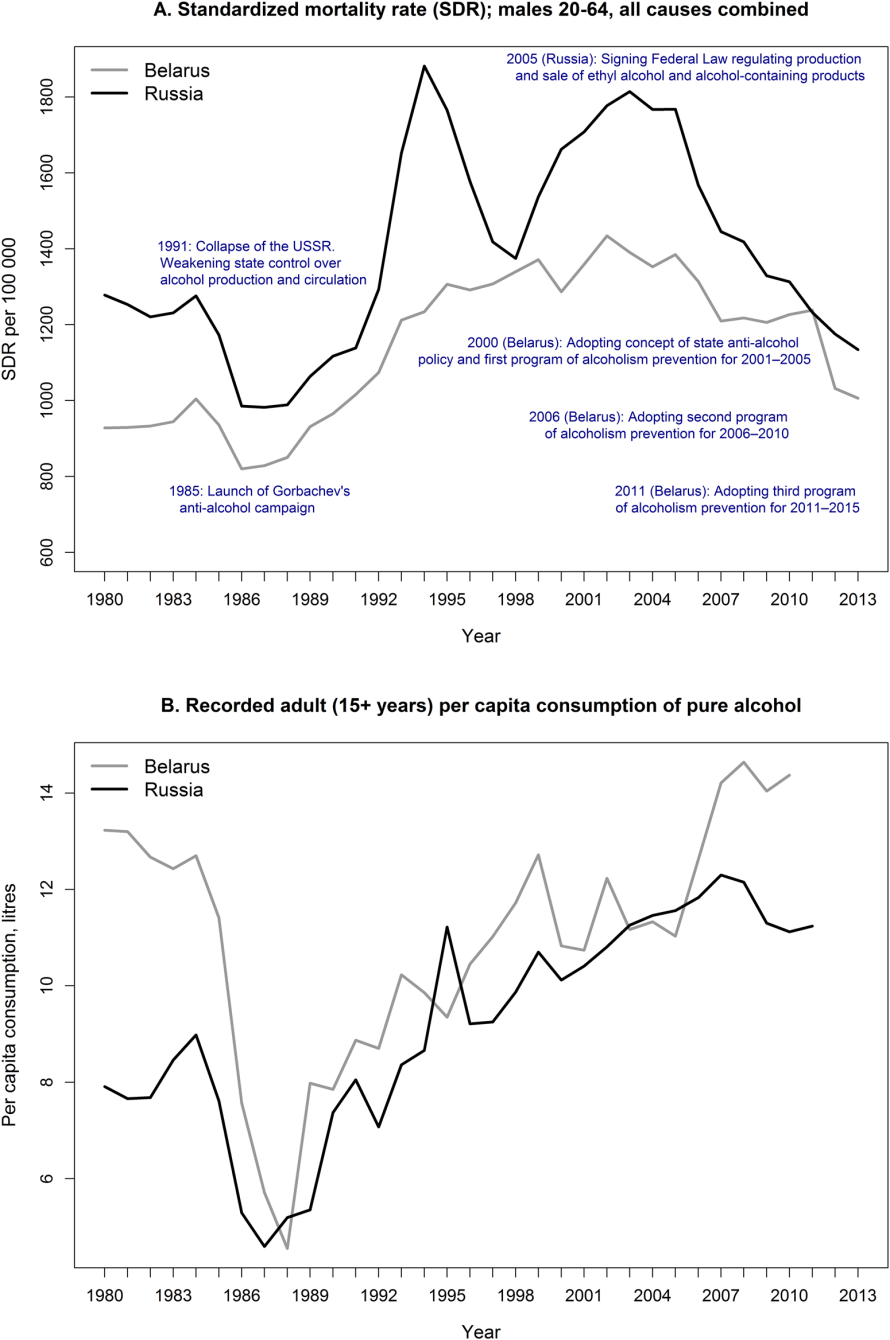
The trends in overall mortality among adult males and recorded adult per capita consumption of pure alcohol; Belarus and Russia, 1980–2013. Source: Panel A: Belarus—until 2010 [34], since 2011, official statistics (unpublished tables); Russia [36]. Panel B: [42].

Fig 1 shows as background information the trends in adult male mortality from all causes of death combined (Panel A) and the dynamics of recorded [42] adult per capita consumption of pure alcohol (Panel B) in Belarus and Russia since 1980.

It can been seen that there was a substantial difference in the overall mortality rates between Belarus and Russia in the yearly 1980s. It is interesting to note that higher mortality observed in Russia at that time corresponds to a much lower level of recorded alcohol consumption. The mortality gap between the two countries narrowed after the launch of the anti-alcohol campaign in 1985 but increased again after the collapse of the USSR in 1991. After quite different developments in the 1990s and early 2000s the trends have finally converged so that the difference between Belarus and Russia in adult male mortality reached its historic minimum.

**Fig 2:**
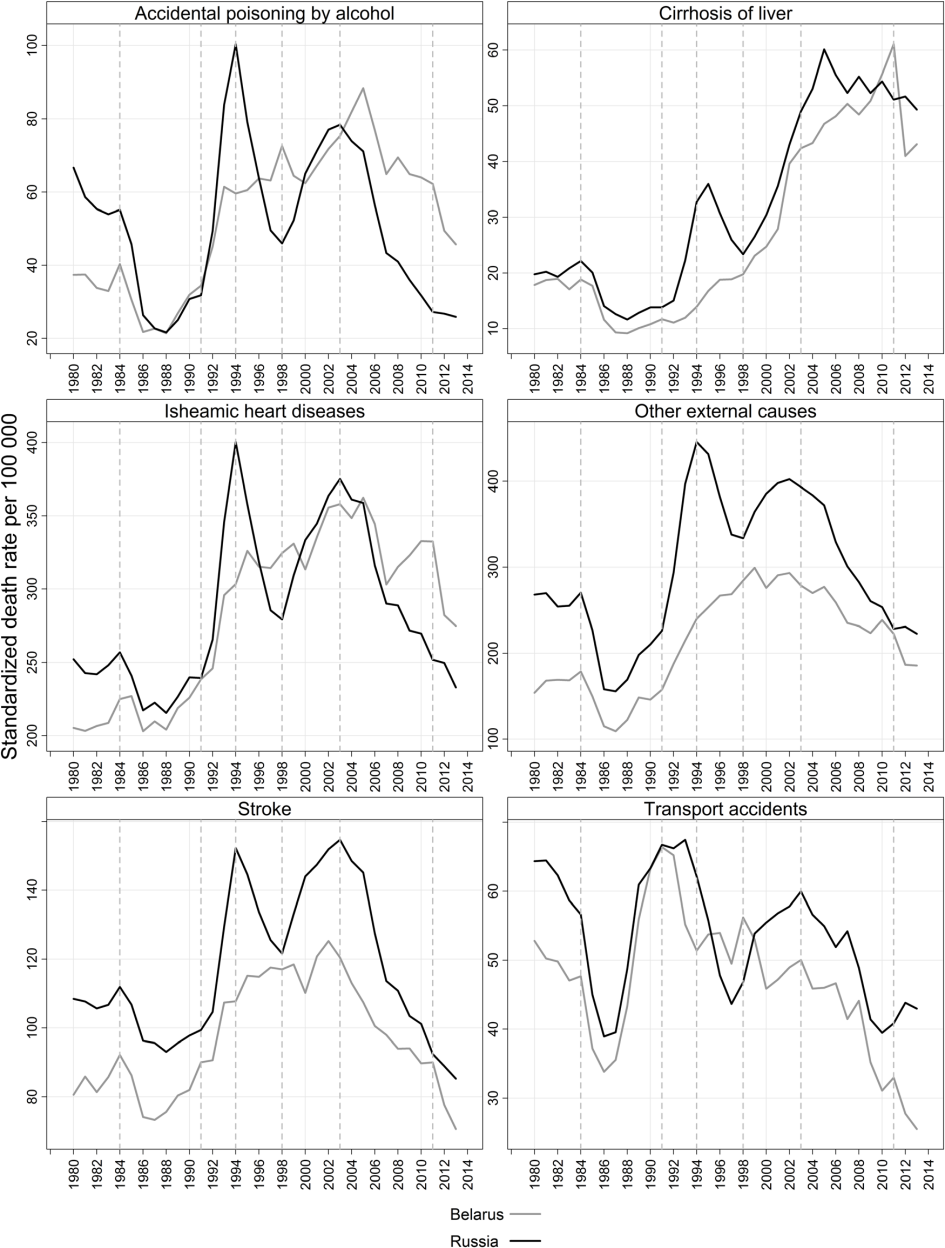
Mortality trends from selected alcohol-related causes of death in Belarus and Russia; males 20–64, 1980–2013. Source: As for Fig 1 (Panel A). Note: Vertical dashed lines represent the major turning points of mortality trends.

The trends in mortality from selected alcohol-related causes appear in Fig 2.

As it is directly associated with the most hazardous forms of alcohol consumption, mortality from accidental poisoning by alcohol is a very sensitive correlate of overall alcohol consumption.[5] Unlike the trends in recorded alcohol consumption the mortality trends from accidental poisoning by alcohol mirror very much the overall mortality trends. Trends in mortality from this cause have fluctuated greatly in Russia, particularly in the 1990s. By contrast, no large fluctuations have been observed in Belarus; instead, mortality in Belarus increased continuously up to 2005, and then began to decline. The improvements in Russia, which started a few years earlier than in Belarus, have been more impressive. Today the levels of mortality from accidental poisoning by alcohol are higher in Belarus than in Russia. This appears to be a reversal of past trends, as historically hazardous forms of alcohol consumption have been more typical of Russia than of Belarus.[43]

The trends in mortality from liver cirrhosis look similar in the two countries. However, in Russia there was a very large increase in the early 1990s, followed by a subsequent decline of about the same magnitude; and, since 1998, by an increase that has been steeper than that of Belarus. In Belarus, mortality from liver cirrhosis increased without interruptions until 2011, when it reached its peak. But in 2012, mortality from liver cirrhosis in Belarus dropped dramatically; by about one-third.

The trends in mortality from ischemic heart diseases are very much in line with those of mortality from accidental poisoning by alcohol, particularly for Russia. Whereas in Russia mortality has been declining rapidly since 2003, in Belarus progress was made starting in 2005, but was quickly interrupted, and did not resume until 2011. Despite a huge decline in 2012, mortality from ischemic heart diseases among adult Belarusian males is currently higher than among their Russian counterparts. While the trends in mortality from stroke have been similar, the pace of the reduction in mortality from stroke has been more impressive and sustainable than the speed of the reduction in mortality from ischemic heart diseases. In both countries, progress has been ongoing since the beginning of the 2000s. In Belarus, it accelerated after 2011. Currently, in both Belarus and Russia mortality from stroke among adult males is at historic lows. The trends in mortality from transportation accidents have also improved, particularly in Belarus, where mortality has been declining steadily since 1990. As was the case with other alcohol-related causes, there was a notable decline in 2012, although by this point in time mortality from transportation accidents had already reached relatively low levels in Belarus.

### Components of the 2012 Mortality Decline in Belarus

Between 2011 and 2012 male life expectancy in Belarus increased by two years.[21]. As such a rapid mortality decline is unusual for contemporary populations, it deserves particular attention. Table 1 summarizes the results of the decomposition analysis The period of sudden mortality decline in Belarus in 2012 (2011–2012) is contrasted to the improvements of similar magnitude observed in Belarus and Russia during the period of the Gorbachev’s anti-alcohol campaign (1984–1987).

**Table 1:** Age-cause components of the change male life expectancy in Belarus (2011–2012 and 1984–1987) and Russia (1984–1987), years.

	Belarus		Russia
	2011–2012	1984–1987	1984–1987
**Change in life expectancy of which by age groups**:	**1.97**	**2.18**	**3.16**
**0**	**0.03**	**0.17**	**0.06**
**1–19**	**0.08**	**0.05**	**0.20**
**20–64**	**1.67**	**1.73**	**2.62**
Accidental poisoning by alcohol	0.11	0.19	0.29
Cirrhosis of the liver	0.17	0.08	0.07
Ischemic heart diseases	0.33	0.11	0.23
Stroke	0.09	0.15	0.11
Transportation accidents	0.06	0.13	0.18
Other external causes	0.34	0.73	1.15
Other causes of death	0.57	0.34	0.59
**65+**	**0.19**	**0.23**	**0.27**

Source: As for Fig 2

The increase in male life expectancy was almost exclusively attributable to rising life expectancy in the population aged 20–64. The reduction in adult mortality from causes which are closely linked to alcohol explains about two-thirds of the improvements. The pattern of mortality reduction observed in Belarus is very similar to the pattern seen in this country and in Russia during Gorbachev’s anti-alcohol campaign. However, unlike in the 1980s, these recent improvements are also linked to reductions in mortality from ischemic heart diseases and from liver cirrhosis. This is largely because these causes of death contribute to mortality levels to a much greater extent currently than they did in the 1980s.

### Mortality reduction and anti-alcohol measures

**Fig 3:**
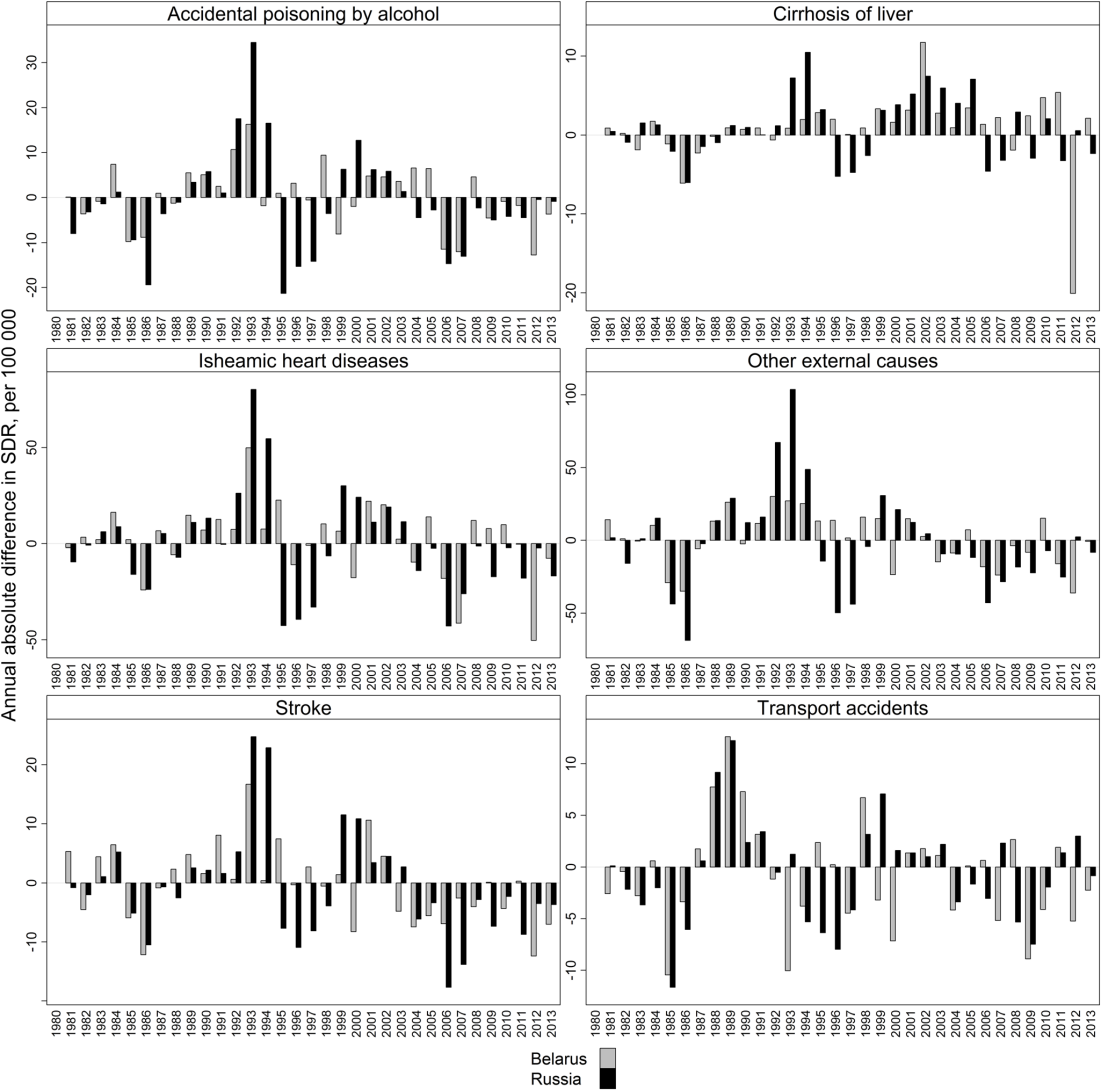
First-order differences in the SDRs from selected alcohol-related causes in Belarus and Russia; males 20–64, 1980–2013. Source: As for Fig 2.

Can the recent decline in adult male mortality in Belarus and Russia be directly attributed to the anti-alcohol policies in these countries, or there is a more complex mechanism behind this phenomenon? If we consider the experience of Gorbachev’s anti-alcohol campaign, we would expect to find that the implementation of anti-alcohol measures would have an immediate impact on mortality. Fig 3 shows the first-order differences in the standardized death rates (SDR) from selected alcohol-related causes in Belarus and Russia since 1980.

The results show that in both countries there were major reductions in mortality in 2006–2007 and in 2012–2013. Russia had more significant declines in 2006–2007, while Belarus saw greater decreases in 2012–2013. The mortality reduction observed in Russia in 2006 can be attributed to the anti-alcohol measures implemented in 2005–2006. But given its magnitude, it is unlikely that the subsequent reduction seen in 2007 is attributable to this factor alone. The 2006–2007 decrease in alcohol-related mortality in Belarus can be linked to the 2006 anti-alcohol campaign. Razvodovsky [44] has provided evidence showing that some of the campaign’s measures were successful. The efforts by the Ministry of Internal Affairs to curb the production of “*samogon*” and the spread of alcohol surrogates led to a substantial decline in the consumption of illegal alcohol. [44] This decline appears to explain the rapid reduction in mortality from accidental poisoning by alcohol observed in Belarus in the middle of the 2000s. It is interesting to note that, unlike other alcohol-related causes, mortality from liver cirrhosis was unchanged in Belarus, and changed only modestly in Russia.

By contrast, a huge decline in mortality from this cause was recorded in Belarus in 2012. There were also marked improvements in mortality from the other alcohol-related causes, such as ischemic heart diseases, stroke, and external causes. This sharp drop in mortality rates can be linked to the most recent anti-alcohol campaign.

## Discussion

### Limitations of the study

Several limitations of this study have to be mentioned. First of all, using a statistical technique such as an Autoregressive Integrated Moving Average (ARIMA) would be an appropriate way to examine statistically the effects of alcohol policies on mortality. [45–48] However, its application is problematic in the settings of the rapid mortality decline observed in Belarus and Russia within a short period of time. The available yearly data do not allow us to come up with reliable statistical inferences. The effective use of the ARIMA would be possible should we have detailed cause-specific monthly or even weekly data at our disposal. This has been already demonstrated by a number of studies on Russia [29–31].

Secondly, an important aspect which this study omits is examining the association between alcohol policies and changes in per capita alcohol consumption. Unfortunately, in the countries considered in the analysis, especially in Russia, official statistics on alcohol consumption are unreliable due to the under-registration of the consumption of illegal alcohol and surrogates [39]. According to the WHO estimates the completeness of the registration of alcohol consumption in Belarus and in Russia is 82 and 76 per cent, respectively. [19] These estimates, however, are likely to be too ‘optimistic’. More importantly, anti-alcohol measures generally assume the improvement of the quality of the registration. That makes official data on alcohol consumption even more implausible (as shown by Fig 1).

Finally, the quality of death registration has to be taken into account while analyzing mortality data by cause. Generally, cause-of-death statistics of adult male population are considered to be reliable and comparable across the FSU countries. [27] Compared to other causes of death the quality of registration of deaths from external causes including accidental poisoning by alcohol is expected to be higher as all of these deaths subject to forensic autopsies. [49]Among ‘natural causes’ of death the quality of the registration of deaths from cardiovascular diseases represents the major concern. [18,50–52]

### Main results

The recent reduction in mortality among adult males in Belarus and Russia is substantial, and shows signs of having lasting effects. Until 2003–2004, there were large fluctuations in these countries in both overall adult mortality and alcohol-related mortality in particular. Gorbachev’s anti-alcohol campaign generated a massive “mortality wave” which seems to still be affecting mortality dynamics. Thus, it is very difficult to assess the impact of policy interventions, and to disentangle their pure mortality effects.

Our results do not lead us to conclude that the schedule of anti-alcohol measures corresponds to the schedule of mortality changes seen in Belarus and Russia. First, in both countries alcohol-related mortality started declining before the major anti-alcohol measures went into effect. Furthermore, given the magnitude of the reductions in alcohol-related mortality observed in Russia in, for example, 2007 and 2010, it is difficult to attribute these declines to anti-alcohol measures. Finally, despite the implementation of anti-alcohol measures, mortality from liver cirrhosis continued to increase in Russia until very recently. In Russia, there was a lag between the decline in mortality from accidental poisoning and the decrease in mortality from liver cirrhosis. In Belarus, mortality from liver cirrhosis continued to increase after the decline in mortality from accidental poisoning had started. This might be explained by the shift in the composition of alcohol consumption towards ‘lighter’ drinks (beer, wine, etc.) as well as changes in drinking habits such as reduction in the prevalence of the most hazardous forms of drinking associated with immediate death. Meanwhile, all these changes could result in increasing damages to the liver among individuals who had already been suffering from chronic liver diseases. On the other hand, the acceleration of the mortality decline in 2006 can indeed be linked to anti-alcohol policies. It has, however, been suggested that this acceleration made only a small contribution to the overall decline.[29,30]

To understand the underlying mechanisms of the recent changes in alcohol-related mortality, it is important to recognize the role of past dynamics. Generally, alcohol-related mortality is unstable, and tends to change over time.[53] In the former USSR, large mortality fluctuations occurred after Gorbachev’s anti-alcohol campaign was implemented. Avdeev and colleagues [54] attributed the increase in mortality in Russia between 1992 and 1994 to the existence of a specific population group who were engaged in the most hazardous forms of drinking. The anti-alcohol campaign of 1985 may have simply postponed the deaths of these individuals; i.e., the size of the group at risk may have increased as a result of the campaign. The relatively large size of the group who regained access to alcohol (particularly cheap and low-quality alcohol) in the early 1990s might explain the mortality epidemics observed among adult males in that period.[54] Subsequent research has also shown that there is a group of high-risk drinkers in Russia [12], and that the dynamics of this group could explain the oscillations in alcohol-related mortality.

We argue that there are two major pathways through which anti-alcohol measures can influence alcohol-related mortality. On one hand, these measures can reduce the overall risk of mortality from spontaneous (unsystematic) drinking, predominantly due to trauma; and from the adverse consequences of a disease not related to alcohol. On the other hand, the anti-alcohol policies can directly influence the dynamics and mortality of the high-risk group. The anti-alcohol measures implemented in the USSR in 1972 are examples of the first pathway. These campaigns did little to reduce mortality, except for having a barely discernible effect on mortality from external causes among young adults. It can be assumed that mortality from spontaneous drinking decreased. At the same time, however, people who drank alcohol regularly expanded the list of liquids they were willing to consume to include items such as cleaning agents. Anecdotal evidence suggests that, until 1985, the following types of daily drinking patterns were prevalent in the USSR: 1) drinking at work, usually at lunchtime, but possibly continuing into the second half of the day; 2) drinking on the way home from work, often near the store where the alcohol was purchased in the "for three" mode; 3) drinking at home after work during lunch/dinner, and later in the evening outside of the house, often in the company of neighbors; and 4) drinking at public events, such as during demonstrations, “*subbotniks*,” collective trips to the park, or walks in nature. It is likely that the very strict measures imposed by the anti-alcohol campaign of 1985 limited drinking to the third type listed above. As a result of the ban on drunkenness at work and on public drinking near shops and at outdoor events, people began to drink less. The amount of alcohol consumed in a single session of drinking was also reduced as it was difficult to get large quantities at one time. As a result, the incidence of hazardous drinking declined. An analysis of causes of death during the anti-alcohol campaign showed that the decline in mortality observed in this period was attributable primarily to a reduction in deaths due to causes common among the group of individuals who engage in hazardous drinking. [4] It thus appears that the tight restrictions on access to alcohol led to decreasing mortality among the incumbent members of this high-risk group, and that the factors mentioned above prevented new members from joining. The rationing of alcohol led to a reduction in the number of “*zapoi*,” a Russian term used to describe a period of two or more days of continuous drunkenness during which the person withdraws from normal social life. According to Nemtsov, [39] the reduction in the frequency of *zapoi* during the anti-alcohol campaign led to a decrease in the number of cases of alcohol dependency and abuse, and generally resulted in a decline in alcohol-related mortality.

However, in the early 1990s all types of drinking re-emerged. In 1992–1994 in Russia (and in 1992–1995 in Belarus and Ukraine) there was an explosion in alcohol-related mortality.[55] Afterwards, mortality started declining. However, this trend was interrupted in Belarus after one year, and it ended in Russia and Ukraine in 1998. Meanwhile, in the Baltic states the speed of the improvement in alcohol-related mortality was slowing, and mortality started to rise again. It is important to note that these fluctuations were not associated with alcohol policies, as there were few such policies in place at that time. The passage in Russia of the Federal Law of 1995, which went into effect in 1996, brought about some superficial changes. After 1998, alcohol-related mortality in Russia began to rise again, reaching a peak in 2003. However, mortality started to decline after 2003, and this trend has lasted for more than a decade.

The anti-alcohol measures implemented in Russia in 2005 and subsequent years did not result in any real declines in the availability of alcohol, except during a short period in the summer of 2006. [28] In all other respects, these measures were more similar to the measures of 1972 than to those of 1985. In the 2000s, the restrictions on the hours during which alcohol may be sold were less extreme than they were in 1972, and the increases in alcohol prices were lower than the increases in 1982. Because they make it more difficult for people to purchase alcohol, restrictions in the hours of sale can lead to reductions in the number of sessions of alcohol consumption and in the amount of alcohol consumed in a single session. It appears, however, that drinkers quickly adapt to new conditions. The price increases in 1982 were somewhat more effective, but only because the campaign also included strict prohibitions on the production of moonshine. The responses to anti-alcohol campaigns appear to have been similar in Belarus, with the possible exception of that country’s 2011 anti-alcohol campaign, which seems to have had different effects. The effects on mortality of these recent campaigns appear to be very similar to those of Gorbachev’s anti-alcohol campaign. It is important to note, however, that the types of actions implemented in these two campaigns are quite different. Even in the context of contemporary Belarus, where the authorities have almost unlimited administrative resources, it is not, for example, feasible to implement rationing. The marked reduction in male mortality observed in Belarus in 2012 is therefore unlikely to be fully attributable to these recent anti-alcohol measures. It appears that the measures of the 2011 campaign coincided with favorable dynamics in alcohol-related mortality. Furthermore, one of the likely preconditions of this large mortality effect is the relatively large size of the group of people who have a much higher alcohol-related mortality risk than the general population. Another possible precondition is the fact that, compared to the previous attempts of Belarusian authorities to combat alcoholism, the measures included in the third anti-alcohol campaign placed much more emphasis on reducing mortality among this high-risk group.

In sum, we believe that the continuous reduction in adult mortality which has been observed in Belarus and Russia over the past decade cannot be explained by the anti-alcohol policies implemented in these countries. Nemtsov and Shelygin [56] who analyzed the reduction in alcohol consumption and anti-alcohol measures implemented in Russia since 2005 came to the similar conclusion. The same authors have also suggested that is hardly possible to disentangle and quantify mortality effects of the alcohol policies, and that there is no appropriate statistical technique to do so. Nevertheless, the anti-alcohol measures introduced in Belarus and Russia in 2005–2006 and in Belarus in 2012 do appear to have had an impact on mortality. However, their effects on mortality have likely been much more modest than some researchers have claimed.[57,58] Scholars who have talked about the effectiveness of anti-alcohol measures have failed to explain the underlying mechanisms of these measures. Soft measures such as increases in the price of alcohol would not stop people who suffer from alcohol dependence from drinking. Such measures may be expected to do little to reduce mortality in the high-risk group, although they might slow the growth in the size of the group. The anti-alcohol measures implemented in Belarus and Russia in the 2000s coincided with fluctuations in alcohol-related mortality which have their roots in the past. If these trends had not been underway already, such large mortality effects would not have been achieved. This implies that a new phase in the expansion of alcohol-related mortality is possible.

## Supporting Information

S1 Appendix List of the major normative acts regulating manufacture, circulation, and consumption of alcohol production adopted in Belarus and Russia during the post-Soviet period (in chronological order). (DOC)
