# Supplementary material for: The Huge Reduction in Adult Male Mortality in Belarus and Russia: Is It Attributable to Anti-Alcohol Measures?
Source: PLoS One. 2015 Sep 16;10(9):e0138021. doi: 10.1371/journal.pone.0138021 (PMC4574310; doi:10.1371/journal.pone.0138021)
Supplement: S1 Appendix — (DOC) [file pone.0138021.s001.doc]

Appendix

List of the major normative acts regulating manufacture, circulation, and consumption of alcohol production adopted in Belarus and Russia during the post-Soviet period (in chronological order)

1. Belarus

| **Date** | **Act** | **Goals/measures/actions** |
| --- | --- | --- |
| 20.07.1998 | Law №193-3: “On the state regulation of the production and circulation of alcohol” | Regulated the licensing of the production, sale, import, and export of alcohol. Established quotas for imports and exports. Regulated prices on alcohol. Set limits on the volume of the alcohol produced, imported, and exported. |
| 03.08.1999 | Presidential Decree №31: “On further measures on the state regulation of the production and circulation of alcohol, non-food ethanol-containing beverages, ethanol produced from non-food raw materials, and tobacco” | Mandated the marking of alcoholic beverages with excise stamps. Required that sellers of alcohol possess special devices for detecting whether alcohol products are genuine. Imposed penalties (termination of license or fines) for violating the decree. |
| 06.01.2000 | Directive of the Council of Ministers: “The concept of the state anti-alcohol policy” | Promoted healthy lifestyles. Mandated improvements in the quality of the prevention, diagnosis, and treatment of alcohol-related diseases. Regulated the production and circulation of alcohol. Mandated a reduction in the production of alcoholic beverages with alcohol content of more than 28%, and an increase in the production of high-quality wines and beers. Banned alcohol advertising, and tightened controls on the quality of alcohol production. Mandated investment in narcology services. |
| 23.08.2000 | Directive of the Council of Ministers №1332: “State program of national alcoholism prevention actions for 2001–2005” |
| 18.12.2002 | Presidential Decree №30: “On the state regulation of the production, circulation, and advertising of alcohol, non-food ethanol-containing production, and ethanol” | Strengthened state control over the production and circulation of alcohol. With reference to Decree 31 (03.08.1999), mandated that licenses for manufacturing be issued by the committee responsible for standardization, meteorology, and certification. Imposed larger fines for violating the decree. |
| 09.09.2005 | Presidential Decree №11: “On the enhancement of state regulation of the production and circulation of alcohol, non-food ethanol-containing production, and ethanol” | Imposed administrative penalties for offenses related to the production and circulation of alcohol and ethanol. |
| 27.04.2006 | Directive of the Council of Ministers №556: “State program of national alcoholism prevention actions for 2006–2010” | Mandated a reduction in per capita alcohol consumption, morbidity, and disability related to excessive alcohol consumption. |
| 10.05.2007 | Law: “On advertisement” | Banned advertisements of alcoholic beverages on radio and television, with the exception of advertisements for beer, which could be aired between 8:00 pm and 7:00 am. Banned the use of statements claiming that consumption of beer is beneficial for individual well-being or success, or that it strengthens physical or mental health. |
| 27.08.2008 | Law: “On the state regulation of the production and circulation of alcohol, non-food ethanol-containing production, and ethanol” | Replaced Law №193-3 (20.07.1998). Imposed bans or limitations on the production and circulation of alcohol, non-food ethanol-containing beverages, and ethanol. |
| 11.01.2011 | Directive of the Council of Ministers №27: “State program of national alcoholism prevention actions for 2011–2015” | Placed more emphasis on the prevention of alcoholism through the reduction in major risk factors. Called for reductions in alcohol-related mortality (mortality rate from accidental poisoning by alcohol), morbidity from cases of alcohol psychosis and dependence. Mandated improvements in narcology services in the country (treatment and prevention of alcoholism). Called for the prevention of and reduction in alcohol-related crimes. Increased the excise duties on specific alcohol products. Increased fines and strengthened police control of home producers. Strengthened laws on drunk driving. |

1. Russia

| **Date** | **Act** | **Goals/measures/actions** |
| --- | --- | --- |
| 22.11.1995 | Federal law №171-FZ: "On the state regulation of production and circulation of ethyl alcohol and alcohol products” | Mandated licensing for the production and sale of alcoholic beverages. Introduced excise marking. Mandated that alcohol (except beer) could only be sold by entities with retail space of not less than 50 square meters. Restricted the ethanol content of alcoholic beverages to 40% by volume, except in the northern regions. Banned the sale of alcohol to children under 18 years old. Banned trade in alcohol in children's, educational, health care facilities, and adjacent areas. Banned trade in beverages with an ethanol content of more than 16.5% in crowded public places, such as transportation hubs. Banned trade in alcohol from 11 pm to 8 am local time. Banned the consumption of alcoholic beverages in public places other than restaurants. Prohibited the production of alcohol from non-food raw materials. |
| 07.03. 2005 | Federal law №11-FZ: “On limits on the retail sale and consumption (drinking) of beer and beverages derived from beer” | Applied the restrictions on spirits to the sale and consumption of beer. |
| 23.05.2005 | Russian Federation Government Decree № 631-p: "On the creation of the state unitary enterprise Rosspirtprom" | Imposed a state monopoly on the production of alcohol. Mandated that Rosspirtprom would control the production of alcohol and set quotas for producers of alcoholic beverages. |
| 21.07.2005 | Federal Law № 102-FZ: "On amendments to the federal law on the state regulation of the production and sale of ethyl alcohol and alcohol products, and the repeal of certain provisions of the federal law on amendments to the federal law on the state regulation of production and sale of ethyl alcohol and alcohol products" | Increased the amount of capital producers of ethanol were required to have, which resulted in a significant reduction in their numbers. Gave regional administrations the right to raise the requirements for trading organizations in the licensing of alcohol retailers (starting in January 2006). Defined a mandatory denaturant for nonbeverage alcoholic liquids which rendered them unfit for drinking (starting in July 2006). Introduced new licenses (starting in July 2006) for the production and wholesale of alcoholic beverages through a new system of excise stamps and a uniform state automated information system for the electronic registration of alcohol (EGAIS). Excluded from the scope of the law changes in activities related to the production and trafficking of beer, as well as of natural beverages with an ethyl alcohol content of less than 6%, including products made from wine which were produced without the addition of ethyl alcohol. |
| 31.12.2008 | Presidential Decree № 1883: "On the formation of the Federal Service for Alcohol Market Regulation" | Mandated that the “Rosalkogolregulirovaniya” would have licensing control over the production and sale of ethyl alcohol and alcohol products (except for retail sales), the power to supervise compliance with the requirements for ethyl alcohol and alcohol products, the power to receive declarations regarding the production and sale of alcohol and alcohol products, the power to issue stamps for alcoholic beverages produced in the territory of Russia, the authority to maintain the registry of licenses for the production and trafficking of alcohol and alcohol products, and the power to maintain the EGAIS. |
| 06.2009 (actually 09.2009) |  | This date marked the start of the operation of the new version of the EGAIS and its gradual transfer to the Rosalkogolregulirovaniya. |
| 14.12.2009 | Directive of the government of the Russian Federation on the approval of a package of measures to improve the efficiency of the market regulation of alcoholic beverages | Established additional restrictions on retail alcohol, including beer and low-alcohol beverages (volume of added ethyl alcohol of less than 7%). Limited the maximum size of a low-alcohol product to 330 ml. Mandated the use of labels (occupying at least 20% of the area of the label) reminding the consumer of the health dangers of alcohol consumption. |
